# Steroid treatment increases the recurrence of radiation-induced organizing pneumonia after breast-conserving therapy

**DOI:** 10.1002/cam4.255

**Published:** 2014-05-03

**Authors:** Keisuke Otani, Kinji Nishiyama, Yuri Ito, Yoshifumi Kawaguchi, Hideo Inaji

**Affiliations:** 1 Department of Radiation Oncology, Osaka Medical Center for Cancer and Cardiovascular DiseasesOsaka, Japan; 2 Department of Cancer Epidemiology and Prevention, Center for Cancer Control and Statistics, Osaka Medical Center for Cancer and Cardiovascular DiseasesOsaka, Japan; 3 Department of Surgery, Osaka Medical Center for Cancer and Cardiovascular DiseasesOsaka, Japan

**Keywords:** Breast cancer, breast-conserving therapy, organizing pneumonia, radiation-induced lung injury, steroid therapy

## Abstract

Radiation-induced organizing pneumonia (RIOP) is an important complication of postoperative radiotherapy for breast cancer. Unfortunately, conventional corticosteroid therapy is frequently associated with relapses. The aim of this retrospective study was to evaluate the outcomes of steroid treatment in patients with RIOP. In total, 26 patients diagnosed with RIOP from among 2404 women who received radiotherapy after breast-conserving surgery for breast cancer were included and classified into steroid (*n* = 7) and nonsteroid (*n* = 19) groups. Serum, sputum, and bronchoalveolar lavage composition; subjective symptoms (cough, fever, and dyspnea); migratory progression; and RIOP relapse were compared between the groups. Treatment type did not affect the duration of the subjective symptoms, which was 1.6 and 1.7 months for the steroid and nonsteroid groups, respectively. In contrast, RIOP relapse and new pulmonary lesions developed in five patients in the steroid group and only three patients in the nonsteroid group (*P* = 0.014). By assessing RIOP duration as the time to resolution of symptoms and discontinuation of therapy, the median duration of RIOP was significantly longer in the steroid (17.1 months) than that in the nonsteroid group (2.3 months, *P* = 0.005), primarily because of frequent relapses. After remission, persistent pulmonary dysfunction did not occur in the nonsteroid group. This single-center retrospective study demonstrates that steroid therapy results in frequent relapses and significantly prolongs RIOP duration. Corticosteroid treatment is considered a critical factor in RIOP recurrence.

## Introduction

Early-stage breast cancer is currently treated by breast-conserving therapy, involving partial mastectomy followed by postoperative radiotherapy of the remaining breast tissue 1. The elaborate focusing on the remaining breast tissues with tangential fields affects a small volume of lung tissues. This complication is characterized by fibrotic changes detected by computed tomography (CT). Occasionally, massive pulmonary infiltrates appear outside the irradiated lung fields 2,3 in a few patients, and this disorder is classified as radiation-induced bronchiolitis obliterans organizing pneumonia (BOOP) syndrome 4. Since bronchiolitis obliterans implies nonessential airway obliteration 5, we introduce the term radiation-induced organizing pneumonia (RIOP) to describe BOOP caused by radiotherapy.

Approximately 2–3% patients develop RIOP after radiation to the residual breast tissues in breast-conserving therapy 4,6–8. Lymphocytic inflammatory reaction to radiation-induced lung injury plays an important role in RIOP development 4,8,9. Accordingly, most RIOP patients receive corticosteroid therapy designed to treat severe radiation pneumonitis (RP) in lung cancer. Once severe RP occurs, the patient's condition often becomes life-threatening despite intensive care and treatment; therefore, steroid administration is recommended for RP. Steroid therapy for RIOP patients has reportedly shown favorable results with no associated mortality, although frequent relapses have recently been reported in association with this therapy 4,7,10–13. Therefore, routine administration of corticosteroids to RIOP patients is debatable. The effectiveness of nonsteroid therapy as an alternative treatment for RIOP remains unclear with regard to the long-term clinical outcomes, presumably because of the limited number of studies. This long-term, single-center study aimed to examine the effectiveness of nonsteroid therapy in preventing RIOP recurrence in comparison with that of steroid therapy.

## Materials and Methods

### Patient selection

This retrospective study was conducted on 2813 women who underwent breast-conserving therapy at our institution from January 1997 to March 2011. Informed consent was obtained from each patient. This study was conducted according to the regulations and ethical policies approved by the institutional review board of our institution.

### Postoperative radiotherapy

A total of 2404 patients received postoperative radiotherapy comprising nonopposed tangential 4- or 6-MV photon beams. The prescribed radiation dose to the whole breast was 50 Gy by a delivery of 2 Gy a day for 5 days in a week. Patients with positive margins received a boost dose of 13.2 Gy in six fractions to the tumor bed (8 × 8 cm^2^) using an electron field (6–12 MeV). The central lung distance (CLD) was <2.5 cm in most patients. Almost all patients received postoperative chemotherapy and/or endocrine therapy. After radiotherapy, all patients underwent physical examination, routine chest radiography, and a blood test every 2 months. CT was performed if infiltrations were detected by chest radiography or if cough or fever was observed during follow-up visits.

### Diagnosis of RIOP

Diagnosis of RIOP was based on the criteria proposed by Crestani et al. 4: (1) history of radiotherapy to the breast within the last 12 months, (2) presence of general and/or respiratory symptoms for ≥2 weeks, (3) detection of pulmonary infiltrates outside the radiation port, and (4) no specific etiology. Asymptomatic patients were also included considering the clinical course. Patients often manifested new migratory lesions over the course of RIOP, similar to the findings of a previous study 4. We investigated these patients from two perspectives: migratory progression, defined as the appearance of secondary lesions before the disappearance or stabilization of the primary pulmonary infiltrate observed at the initial presentation; and RIOP relapse, identified by the appearance of new pulmonary infiltrates after the disappearance of the initial infiltrate. The diagnosis of RIOP is sometimes difficult to distinguish from normal pneumonia. We have carefully diagnosed RIOP after the bacterial culture or empirical antibiotic therapy. Patients with persistent migratory lung infiltrate were also diagnosed as having RIOP.

### Statistical analysis

Statistical analysis was conducted using Fisher's exact test and a two-sample Wilcoxon rank-sum (Mann–Whitney) test. Statistical analyses were performed using the standard statistical package Stata 12.1 (StataCorp., College Station, TX). Statistical significance was defined as *P *<* *0.05.

## Results

### RIOP patients

A total of 26 patients (1.1%; Table1, Table S1 for details) were diagnosed with RIOP on the basis of the criteria proposed by Crestani et al. 4. Four asymptomatic patients were diagnosed on the basis of routine chest radiography. These patients were 38–77 years old (median age, 59 years). Eight patients were premenopausal and 18 were postmenopausal. Eleven patients reported a history of food or drug allergy. Three patients were ex-smokers. One patient was on oral prednisolone therapy for bronchial asthma (Case 7). The median follow-up period was 41.2 months (range, 3.7–161 months) after the completion of radiotherapy. Ten patients had cancer of the right breast and 16 had cancer of the left breast (no patients had bilateral lesions). The clinical stages were cT1-2N0-2M0, stage I–IIIA according to staging classification standards established by the Union for International Cancer Control (UICC) 7th edition 14. Invasive ductal carcinoma was observed in 19 patients, ductal carcinoma in situ in five, and invasive lobular carcinoma in two. Upon follow-up, recurrence of breast cancer was detected in the axillary lymph nodes from the two living patients. The other 24 patients showed no evidence of recurrence at the end of the follow-up period.

**Table 1:** Patients in the steroid and nonsteroid group.

	Median age	Migratory progression	Relapse^1^ (repeated)	Total patients with New lesions	Duration of RIOP^3^ (months)
Steroid group (*n* = 7)	56	3	5 (3)	6	17.1
Nonsteroid group (*n* = 19)	60	6	3 (1^2^)	9	2.3

We defined migratory progression as the appearance of secondary lesions before the disappearance or stabilization of the primary pulmonary infiltrate observed at the initial presentation, and relapse as identified by the appearance of new pulmonary infiltrates after the disappearance of the initial infiltrate.

1 Fisher's exact test: *P *= 0.014.

2 Patient who started steroid at the first relapse.

3 Time from onset to be free from symptoms, steroid, and other medications.

All patients underwent partial mastectomy; 11 underwent axillary lymphadenectomy and the remaining 15 underwent sentinel lymph node biopsy that showed negative results. The median time from surgery to the start of radiotherapy was 1.5 months. Twenty-four patients received radiotherapy to the residual breast tissues and two received radiotherapy to both the residual breast and ipsilateral supraclavicular fossa. Concurrent systemic therapy included an aromatase inhibitor (AI) in five patients, tamoxifen (TAM) in six, and tegafur–uracil in one. The total absorbed dose with 4- or 6-MV X-rays was 50 Gy in every patient except one; the dose in this one patient was 46 Gy because of chemotherapy-induced progressive leukocytopenia. Four patients received an additional electron boost of 8.4–13.2 Gy in four to six fractions. The median CLD of the fields was 2.1 cm (range, 1.3–3.8 cm). After completion of postoperative radiotherapy, four patients underwent chemotherapy, 17 underwent endocrine therapy (TAM/AI: 8/9), and five underwent routine observations.

### Development of RIOP symptoms and related findings

Table S1 shows the clinical characteristics of the 26 patients that were relevant to this study. In most patients (23/26), RIOP became clinically evident within 6 months after radiotherapy (median: 3.1 months, range: 1.0–12). Subjective symptoms at the first presentation were dry cough in 62% (16/26) patients, productive cough in 23% (6/26), fever (37.7–38.9°C) in 42% (11/26), and dyspnea on exertion in 27% (7/26). Laboratory data showed leukocytosis (9000–10,000/mm^3^) in 12% (3/26) patients. C-reactive protein was positive (2.3–23) in 58% (15/26) patients and negative in 15% (4/26). Bacterial cultures of sputum obtained from nine patients were negative. Typically, chest CT showed coexisting dense infiltrates and ground-glass opacities outside the radiation fields, accompanied by ordinary fibrotic elements inside the radiation fields. The involved lung was ipsilateral in all 26 patients, and additional contralateral lung involvement was observed in six of these patients. Three patients underwent bronchoalveolar lavage, and bacterial culture revealed lymphocytosis and negative results for all patients.

### Treatment of RIOP symptoms

Although corticosteroids were used for the treatment of RIOP at our institution until 2001, we changed this strategy to avoid the routine use of corticosteroids, although patients with severe dyspnea were an exception. Seven patients who were treated before 2001 or showed relatively severe symptoms were administered corticosteroids such as prednisolone (60 mg/day, PO) as the initial treatment; these were classified into the steroid group (Table1). Of these seven patients, three received steroid therapy immediately after RIOP diagnosis. Another three received steroid therapy because they showed refractory symptoms. The remaining one patient was already receiving corticosteroids (Case 7); the dose was increased in this patient. In the steroid group, the interval between the onset and administration of corticosteroids ranged from 0.4 to 3.1 months. The remaining 19 patients received nonsteroid drugs such as Non-Streroidal Anti-Inflammatory Drugs, antibiotics, and antitussives as the initial treatment; these were classified into the nonsteroid group. Four asymptomatic patients in this group were observed without the administration of any specific drugs. These two groups were statistically evaluated if they were balanced with regard to age; history of chemotherapy or endocrine therapy; clinical and pathological 14 stage, T stage, and N stage; and estrogen receptor (ER), progesterone receptor (PR), and Her2 status, and the results revealed that they were balanced in the steroid and nonsteroid groups (Table S2).

### Treatment outcome in the steroid and nonsteroid RIOP groups

In the steroid group (*n* = 7), the subjective symptoms disappeared within a median period of 1.6 months (range, 1.1–2.6 months) after initial presentation and within a median period of 0.2 months (range, 0.1–0.5 months) after the initiation of steroid therapy. In the nonsteroid group (*n* = 15), the subjective symptoms disappeared within a median period of 1.7 months (range, 0.5–6.7 months) after initial presentation, which was comparable with that for the steroid group (Wilcoxon test: *P *=* *0.56). However, five patients (26%) in the nonsteroid group had persistent symptoms for >3 months. The subjective symptoms were resolved before the disappearance or stabilization (scar change) of pulmonary infiltrates in both the groups. Among these symptoms, cough was the last to disappear.

### Migratory progression and RIOP relapse

In this study, 15 patients manifested new migratory lesions over the course of RIOP. New lesions indicative of migratory progression or relapse developed in 86% (6/7) patients in the steroid group and 47% (9/19) patients in the nonsteroid group (Fisher's exact test: *P *=* *0.18; Table1). Migratory progression was identified in three patients in the steroid group (43%) within a median period of 0.7 months (range: 0.2–3.1) after onset and six patients (31.6%) in the nonsteroid group. In the steroid group, migratory progression developed before the onset of steroid therapy, but not after steroid therapy. In contrast, RIOP relapse was observed in 71% (5/7) patients in the steroid group compared with only 16% (3/19) patients in the nonsteroid group (Fisher's exact test: *P *=* *0.014; Table1). Two patients with relapse in the steroid group experienced migratory progression at the first appearance of RIOP; this was not observed in any of the three patients with relapse in the nonsteroid group. In the steroid group, four patients developed relapse during the steroid tapering period, while one developed relapse after completion of steroid treatment. These five patients were treated with steroids on the basis of respiratory symptoms, and three of these patients experienced repeated relapses. Of the three patients who experienced relapse in the nonsteroid group, one who was treated with steroids for severe respiratory symptoms at relapse experienced two relapses. However, the other two patients who were maintained only on nonsteroid therapy experienced no further relapses. Collectively, in the steroid group, the overall median duration of steroid treatment was 7.8 months (range: 1.1–158 months). The chest radiographs showed evidence of complete recovery from pulmonary infiltrates in 19/26 patients, despite the presence of persistent fibrotic scars in six (the other one was unknown). The disappearance of pulmonary infiltrates (or stable fibrosis) was observed in a median period of 7.5 months (range: 1.2–22.9 months) and 7.7 months (range: 1.9–131) after RIOP onset in the nonsteroid and steroid groups, respectively (Wilcoxon test: *P *=* *0.95).

With regard to other factors that may have affected relapse, such as age; history of endocrine therapy or chemotherapy; clinical and pathological TNM stage, T stage, and N stage; and ER, PR, and Her2 status, none of the factors revealed a significant difference (Table S3). Because of our limited sample size, multifactor analysis could not be performed to control the confounding factors between the two groups; however, the depicted factors were similarly distributed.

### Comparison of RIOP duration between the steroid and nonsteroid groups

The duration of RIOP was measured from the onset of symptoms until the complete resolution of symptoms and termination of steroid therapy or the other therapies. The following five patients were excluded from the analysis: one patient from the steroid group with preexisting bronchial asthma (Case 7) and four asymptomatic patients from the nonsteroid group. The median RIOP duration was 17.1 months (range: 4–160 months) in the steroid group (*n* = 6) compared with only 2.3 months (range: 0.5–29.6 months) in the nonsteroid group (*n* = 15, *P *=* *0.005). The duration of RIOP exceeded 2 years in all patients with repeated relapses (Table1). The significant difference in RIOP duration between the two groups was primarily because of the frequent relapses experienced by patients in the steroid group.

### Impact of steroid and nonsteroid therapies on respiratory function

**Figure 1:**
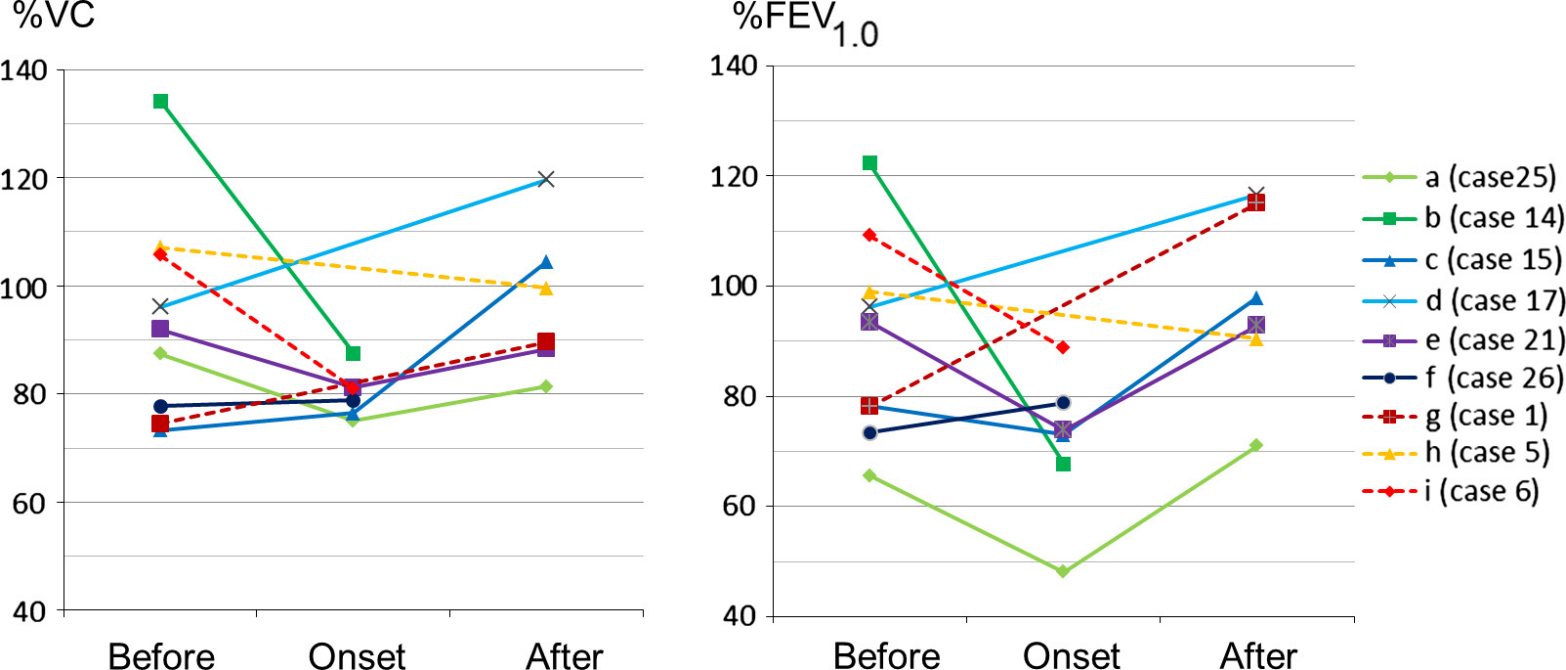
Respiratory function during and after RIOP. (a–e) nonsteroid group; (f) patient in nonsteroid group who began steroid therapy after relapse (case 26); (g–i): steroid group.

Respiratory function tests were performed in several RIOP patients during and/or after RIOP (Fig.1). A patient diagnosed with obstructive lung disease before RIOP (Case 25) exhibited mixed respiratory dysfunction. Another patient with normal function (Case 14) before the onset of RIOP developed obstructive dysfunction. One patient (Case 6) developed a grade 3 respiratory disorder (Common Terminology Criteria for Adverse Events version 4.0) 15 during RIOP. However, the remaining patients showed grade ≤2 respiratory disorder. Respiratory function data were not markedly impaired with regard to both %VC (vital capacity) and %FEV_1.0_ (forced expiratory volume) in most patients after remission (Fig. S1).

### Review of the literature on RIOP

We collected clinical studies on RIOP. The total number of RIOP patients documented in the literature recently reached 100, with 74 reported from Japanese institutions and 26 from Western countries (Table2) 2–4,6,7,10–13,16–19. Patients, whose onset was within 6 months and patients with migratory progression, relapse and new lesions were counted. Assumed duplication is shown in brackets and not summed in total number. To the best of our knowledge, this includes the largest patient sample from a single institution or hospital.

**Table 2:** Reports of radiation-induced organizing pneumonia.

	Number	Onset within 6 months	Patients with migratory progression	Patients with relapse (steroid, nonsteroid)	Patients with new lesions
Reports from Western countries					
Crestani et al. 4	15	NA	14/15	12/15 (12/15, NA)	14/15
Stover et al. 10	1	1	NA	1/1 (1/1, NA)	1/1
Arbetter et al. 11	6	5	1/6	3/6 (3/3, 0/3)	2/2
Cornelissen et al. 17	2	2	NA	2/2 (2/2, NA)	2/2
van Laar et al. 19	2	2	2/2	1/2 (0/1, 1/1)	2/2
Reports from Japan					
Katayama et al. 6	16	13/16	NA	8/16 (NA, NA)	8/16
Ogo et al. 7	37	33/37	NA	[5/32 (NA, NA)]	[5/32]
Ogo et al. 13	[12]	[9/12]	5/12	0/12 (NA, 0/12)	5/12
Kubo et al. 12	12	10/12	NA	4/12 (4/7, 0/5)	4/12
Miwa et al. 16	5	4/5	5/5	2/5 (2/5, NA)	5/5
Takigawa et al. 18	4	4/4	NA	4/4 (4/4, NA)	4/4
Total (except ours)	100	77/88 (88%)	27/38	29/61 (28/41, 1/20)	45/95
Current study	26	23/26 (89%)	9/26	8/26 (5/7, 3/19)	15/26

Assumed duplication is shown in [] and not summed in total number. NA, Not available.

## Discussion

Clinical studies on RIOP are scarce and largely limited to case reports. Our report demonstrated that 89% of the 26 patients were diagnosed with RIOP within 6 months after radiotherapy, which is consistent with the results of previous studies (Table2). This finding emphasizes the importance of monitoring the development of RIOP in breast cancer patients receiving radiotherapy, particularly during the first 6 months.

Corticosteroids constitute the mainstay of treatment for RIOP in patients with breast cancer 4,18. In this study, the steroid group received the same steroid therapy that we routinely use for the effective treatment of RP at our institution. However, we were unclear about the efficacy of steroid therapy for RIOP compared with that for RP in terms of the better prognosis; therefore, we ceased the routine use of corticosteroids for RIOP in 2001, although patients with severe respiratory symptoms were an exception. This study describes our experience with steroid and nonsteroid therapies for RIOP patients cumulated over more than a decade.

Corticosteroids rapidly resolve the symptoms of RIOP in patients with breast cancer. Crestani et al. reported that RIOP symptoms disappeared within 1 week and that the infiltrative shadows diminished within approximately 2 weeks after the onset of prednisone administration 4. Takigawa et al. also reported the rapid improvement of RIOP symptoms after steroid therapy 18. Our report shows that RIOP symptoms resolved over a similar time period after initial presentation in the steroid and nonsteroid groups (median, 1.6 vs. 1.7 months, respectively). However, patients in the steroid group took less than half a month to achieve symptom relief after the administration of steroids. In contrast, some patients in the nonsteroid group experienced persistent symptoms for up to 3 months. Taken together, these data promote steroid therapy for the rapid relief of initial RIOP symptoms.

RIOP is characteristically accompanied by migratory and relapsing pulmonary infiltrates 4. We defined their pattern as migratory progression if they appeared before the remission of pulmonary infiltrates and relapse if they appeared after the disappearance of infiltrates. Migratory progression developed in 93.3% (14/15) patients in the study by Crestani et al. and in all four patients in the study by Miwa et al. 4,16. In this study, migratory progression appeared in 43% and 32% patients in the steroid and nonsteroid groups, respectively. The difference in incidence among the reports may be related to the frequency of radiographic examinations. On the other hand, we report that migratory progression developed prior to steroid administration and was not observed after steroid administration in all patients, as indicated in these twostudies 4,16. Therefore, steroid treatment may also be effective for the suppression of migratory progression.

This study demonstrates that corticosteroid therapy fosters RIOP relapse. Most patients in our steroid group experienced frequent relapses (71%) during steroid tapering or withdrawal. Crestani et al. reported relapse in 80% (12/15) patients treated with steroid therapy (four during steroid tapering) 4. In other reports, patients treated with steroids were reported to relapse frequently (82%, Table2). Takigawa et al. reported relapses in all four RIOP patients treated with steroids 18. Among the studies reported from Japanese institutions, the relapse rate was 63% in 16 patients with RIOP treated by steroids (Table2). Furthermore, Ogo et al. 13. and Kubo et al. 12. reported no relapse among 17 RIOP patients treated without steroids. Arbetter et al. 11. also described that relapse developed in only 3 patients treated with steroids. Van Laar et al. 19. reported a contradictory case with RIOP relapse despite nonsteroid therapy. Because relapse was commonly observed in patients treated with steroids and infrequently in those treated without steroids, RIOP duration was significantly longer in the steroid group, which included a greater number of patients with relapses (*P *=* *0.005).

Repeated relapses are particularly problematic in RIOP patients because they dramatically prolong symptoms and lung complications. Repeated relapses have been reported in 50%–59% RIOP patients receiving therapy 4,18. These data are consistent with those of this study, which demonstrated repeated relapses in 60% patients in the steroid group. In contrast, only one patient in the nonsteroid group exhibited repeated relapses, and she underwent steroid therapy after the first relapse. Overall, RIOP duration exceeded 2 years in four patients who experienced repeated relapses in this study.

Given that no new lesion was recognized during steroid administration, we assume that steroid therapy effectively suppresses the development of migratory progression. However, this suppressive effect seemed to decline with steroid tapering; therefore, new lesions (relapses) particularly appeared in patients in the steroid group. This could be because of two reasons. First, steroids can postpone the development of new lesions by suppressing tissue responses to inflammation during steroid administration. Second, they can cause the relapse of tissue damaging responses. It is necessary to investigate the effects of steroid therapy on RIOP in more detail with more clinical cohorts.

Persistent complications may raise concerns with regard to pulmonary function. However, most studies only reported mild and temporary pulmonary dysfunction in RIOP patients treated with corticosteroids. For example, Crestani et al. reported mild restrictive ventilatory defects in 20% RIOP patients treated with steroids 4. Miwa et al. reported normal respiratory function in all five patients treated with steroids during the active phase of RIOP 16. In these reports, whether RIOP treatment without steroids resulted in persistent pulmonary dysfunction remained unclear. In this study, respiratory examination revealed mild respiratory dysfunction in twopatients during RIOP (steroid/nonsteroid group: 0/2). Nonetheless, all patients recovered normal pulmonary function after RIOP remission. Persistent pulmonary symptoms were not observed in any patient in this study.

This retrospective analysis of a rare disorder is associated with inherent limitations, including the information available in the patient files and the small sample size. Furthermore, the examination or treatment of RIOP was not systematic. A histological diagnosis was not available for all patients; nonetheless, the diagnosis of each RIOP patient was confirmed by the relatively specific criteria established by Crestani et al. 4 on the basis of CT data and medical history. All the symptomatic patients in this study fulfilled these diagnostic criteria.

In conclusion, this study demonstrates that nonsteroid therapies are associated with less frequent relapses and shorter symptom duration without the development of persistent pulmonary complications in RIOP patients. This study suggests that steroid therapy should be avoided to decrease the burden of RIOP in breast cancer patients after breast-conserving therapy.

## Conflict of Interest

None declared.

## Supporting Information

**Figure S1.** Recovery of respiratory function. The respiratory function tests for determining %VC and% FEV_1.0_ were performed either during the onset and/or after the recovery in nine RIOP patients. Each mark shows the individual patient as indicated.

**Table S1.** Clinical characteristics of the patients.

**Table S2.** Patient properties of steroid and nonsteroid groups.

**Table S3.** Evaluation of parameters and RIOP relapse.

## References

[b1] Fisher B, Anderson S, Bryant J, Margolese RG, Deutch M, Fisher ER (2002). Twenty-year follow-up of a randomized trial comparing total mastectomy, lumpectomy, and lumpectomy plus irradiation for the treatment of invasive breast cancer. N. Engl. J. Med.

[b2] Crestani B, Kambouchner M, Soler P, Crequit J, Brauner M, Battesti J-P (1995). Migratory bronchiolitis obliterans organizing pneumonia after unilateral radiation therapy for breast carcinoma. Eur. Respir. J.

[b3] Bayle JY, Nesme P, Bejui-Thivolet F, Loire R, Guerin JC, Cordier JF (1995). Migratory organizing pneumonitis “primed” by radiation therapy. Eur. Respir. J.

[b4] Crestani B, Valeyre D, Roden S, Wallaert B, Dalphin JC, Cordier JF (1998). Bronchiolitis obliterans organizing pneumonia syndrome primed by radiation therapy to the breast. The groupe d'etudes et de recherche sur les maladies orphelines pulmonaires (germ”o”p). Am. J. Respir. Crit. Care Med.

[b5] American Thoracic Society/European Respiratory Society International Multidisciplinary Consensus Classification of the Idiopathic Interstitial Pneumonias (2002). This joint statement of the American Thoracic Society (ATS), and the European Respiratory Society (ERS) was adopted by the ATS board of directors and by the ERS Executive Committee, June 2001. Am. J. Respir. Crit. Care Med.

[b6] Katayama N, Sato S, Katsui K, Takemoto M, Tsuda T, Yoshida A (2009). Analysis of factors associated with radiation-induced bronchiolitis obliterans organizing pneumonia syndrome after breast-conserving therapy. Int. J. Radiat. Oncol. Biol. Phys.

[b7] Ogo E, Komaki R, Fujimoto K, Uchida M, Abe T, Nakamura K (2008). A survey of radiation-induced bronchiolitis obliterans organizing pneumonia syndrome after breast-conserving therapy in Japan. Int. J. Radiat. Oncol. Biol. Phys.

[b8] Prakash UB (1999). Radiation-induced injury in the “nonirradiated” lung. Eur. Respir. J.

[b9] Martin C, Romero S, Sanchez-Paya J, Massuti B, Arriero JM, Hernandez L (1999). Bilateral lymphocytic alveolitis: a common reaction after unilateral thoracic irradiation. Eur. Respir. J.

[b10] Stover DE, Milite F, Zakowski M (2001). A newly recognized syndrome–radiation-related bronchiolitis obliterans and organizing pneumonia. A case report and literature review. Respiration.

[b11] Arbetter KR, Prakash UB, Tazelaar HD, Douglas WW (1999). Radiation-induced pneumonitis in the “nonirradiated” lung. Mayo Clin. Proc.

[b12] Kubo A, Osaki K, Kawanaka T, Furutani S, Ikushima H, Nishitani H (2009). Risk factors for radiation pneumonitis caused by whole breast irradiation following breast-conserving surgery. J. Med. Invest.

[b13] Ogo E, Komaki R, Abe T, Uchida M, Fujimoto K, Suzuki G (2010). The clinical characteristics and non-steroidal treatment for radiation-induced bronchiolitis obliterans organizing pneumonia syndrome after breast-conserving therapy. Radiother. Oncol.

[b14] Sobin L, Gospodarwicz M, Wittekind C (2009). TNM classification of malignant tumours.

[b15] U.S. Department of Health and Human Services, National Institutes of Health, National Cancer Institute (2009). http://evs.nci.nih.gov/ftp1/CTCAE/.

[b16] Miwa S, Morita S, Suda T, Suzuki K, Hayakawa H, Chida K (2004). The incidence and clinical characteristics of bronchiolitis obliterans organizing pneumonia syndrome after radiation therapy for breast cancer. Sarcoidosis Vasc. Diffuse Lung Dis.

[b17] Cornelissen R, Senan S, Antonisse IE, Liem H, Tan YKY, Rudolphus A (2007). Bronchiolitis obliterans organizing pneumonia (BOOP) after thoracic radiotherapy for breast carcinoma. Radiat. Oncol.

[b18] Takigawa N, Segawa Y, Saeki T, Kataoka M, Ida M, Kishino D (2000). Bronchiolitis obliterans organizing pneumonia syndrome in breast-conserving therapy for early breast cancer: radiation-induced lung toxicity. Int. J. Radiat. Oncol. Biol. Phys.

[b19] van Laar JM, Holscher HC, van Krieken JH, Stolk J (1997). Bronchiolitis obliterans organizing pneumonia after adjuvant radiotherapy for breast carcinoma. Respir. Med.

