# Supplementary figures and images for: Steroid treatment increases the recurrence of radiation-induced organizing pneumonia after breast-conserving therapy
Source: Cancer Med. 2014 May 3;3(4):947–53. doi: 10.1002/cam4.255 (PMC4303162; doi:10.1002/cam4.255)

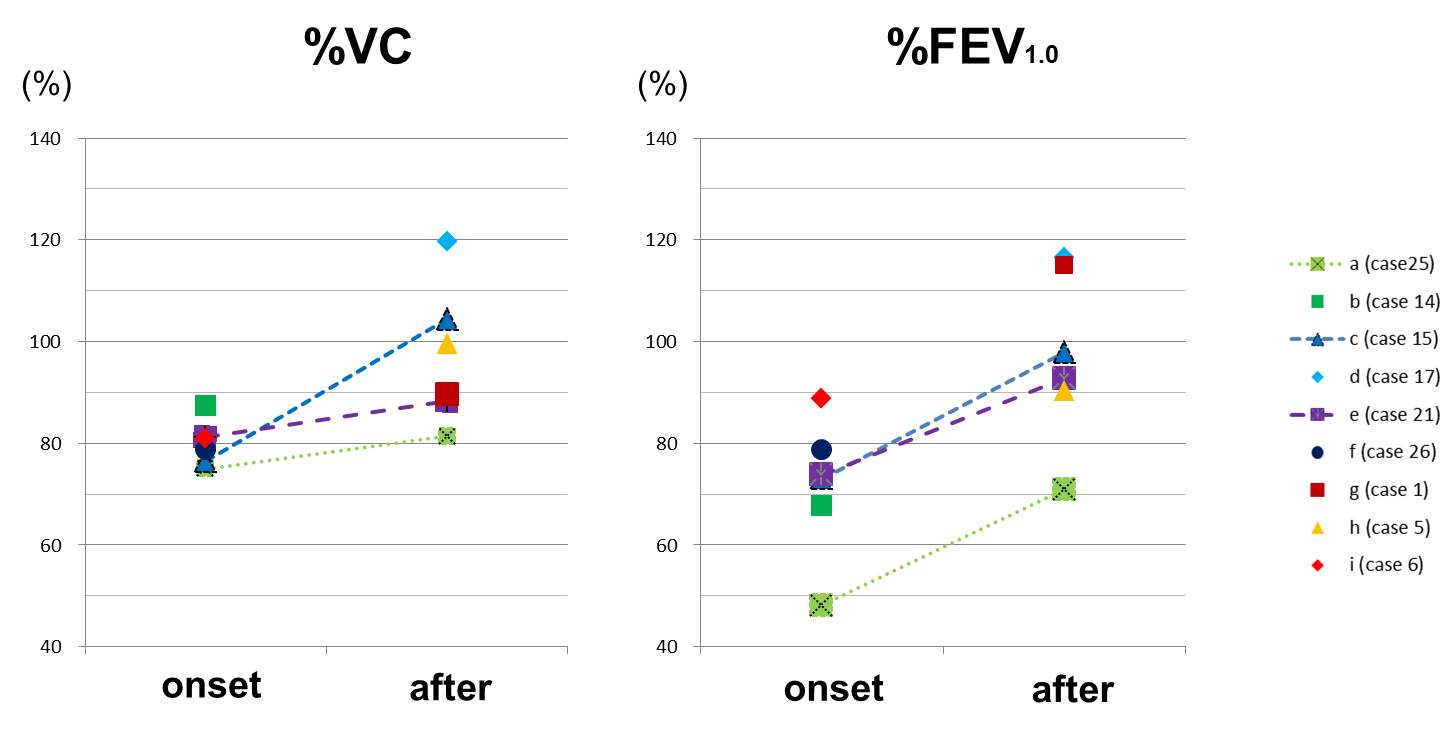

Supplement: Supplementary file 1 [file cam40003-0947-sd1.jpg]
