# Supplementary material for: Steroid treatment increases the recurrence of radiation-induced organizing pneumonia after breast-conserving therapy
Source: Cancer Med. 2014 May 3;3(4):947–53. doi: 10.1002/cam4.255 (PMC4303162; doi:10.1002/cam4.255)
Supplement: Supplementary file 2 [file cam40003-0947-sd2.pptx]

## Slide 1
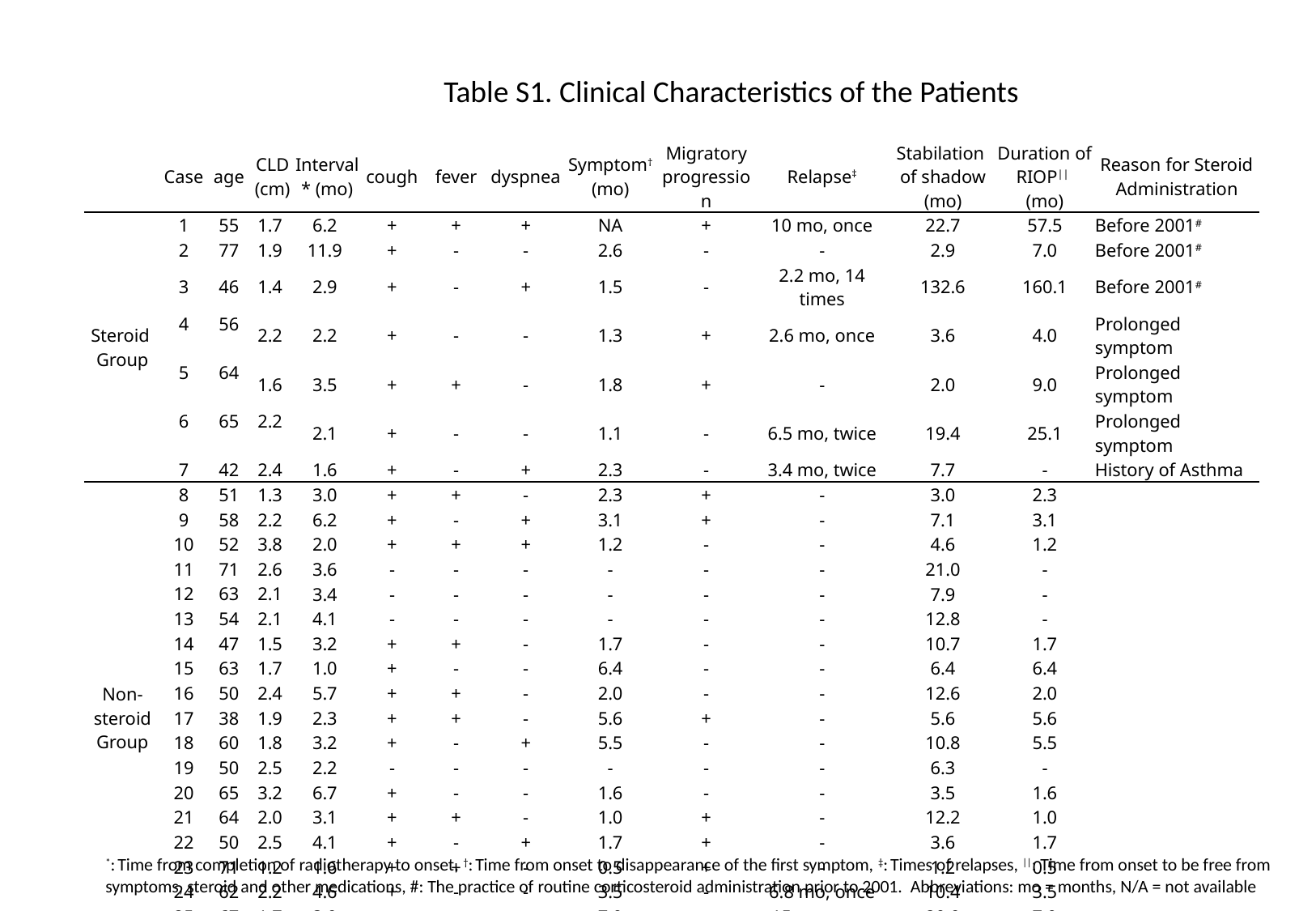

Table S1. Clinical Characteristics of the Patients
| | Case | age | CLD (cm) | Interval\* (mo) | cough | fever | dyspnea | Symptom† (mo) | Migratory progression | Relapse‡ | Stabilation of shadow (mo) | Duration of RIOP|| (mo) | Reason for Steroid Administration |
| --- | --- | --- | --- | --- | --- | --- | --- | --- | --- | --- | --- | --- | --- |
| Steroid Group | 1 | 55 | 1.7 | 6.2 | + | + | + | NA | + | 10 mo, once | 22.7 | 57.5 | Before 2001# |
| | 2 | 77 | 1.9 | 11.9 | + | - | - | 2.6 | - | - | 2.9 | 7.0 | Before 2001# |
| | 3 | 46 | 1.4 | 2.9 | + | - | + | 1.5 | - | 2.2 mo, 14 times | 132.6 | 160.1 | Before 2001# |
| | 4 | 56 | 2.2 | 2.2 | + | - | - | 1.3 | + | 2.6 mo, once | 3.6 | 4.0 | Prolonged symptom |
| | 5 | 64 | 1.6 | 3.5 | + | + | - | 1.8 | + | - | 2.0 | 9.0 | Prolonged symptom |
| | 6 | 65 | 2.2 | 2.1 | + | - | - | 1.1 | - | 6.5 mo, twice | 19.4 | 25.1 | Prolonged symptom |
| | 7 | 42 | 2.4 | 1.6 | + | - | + | 2.3 | - | 3.4 mo, twice | 7.7 | - | History of Asthma |
| Non-steroid Group | 8 | 51 | 1.3 | 3.0 | + | + | - | 2.3 | + | - | 3.0 | 2.3 | |
| | 9 | 58 | 2.2 | 6.2 | + | - | + | 3.1 | + | - | 7.1 | 3.1 | |
| | 10 | 52 | 3.8 | 2.0 | + | + | + | 1.2 | - | - | 4.6 | 1.2 | |
| | 11 | 71 | 2.6 | 3.6 | - | - | - | - | - | - | 21.0 | - | |
| | 12 | 63 | 2.1 | 3.4 | - | - | - | - | - | - | 7.9 | - | |
| | 13 | 54 | 2.1 | 4.1 | - | - | - | - | - | - | 12.8 | - | |
| | 14 | 47 | 1.5 | 3.2 | + | + | - | 1.7 | - | - | 10.7 | 1.7 | |
| | 15 | 63 | 1.7 | 1.0 | + | - | - | 6.4 | - | - | 6.4 | 6.4 | |
| | 16 | 50 | 2.4 | 5.7 | + | + | - | 2.0 | - | - | 12.6 | 2.0 | |
| | 17 | 38 | 1.9 | 2.3 | + | + | - | 5.6 | + | - | 5.6 | 5.6 | |
| | 18 | 60 | 1.8 | 3.2 | + | - | + | 5.5 | - | - | 10.8 | 5.5 | |
| | 19 | 50 | 2.5 | 2.2 | - | - | - | - | - | - | 6.3 | - | |
| | 20 | 65 | 3.2 | 6.7 | + | - | - | 1.6 | - | - | 3.5 | 1.6 | |
| | 21 | 64 | 2.0 | 3.1 | + | + | - | 1.0 | + | - | 12.2 | 1.0 | |
| | 22 | 50 | 2.5 | 4.1 | + | - | + | 1.7 | + | - | 3.6 | 1.7 | |
| | 23 | 71 | 1.2 | 1.6 | + | + | - | 0.5 | + | - | 1.2 | 0.5 | |
| | 24 | 62 | 2.2 | 4.6 | + | - | - | 3.5 | - | 6.8 mo, once | 10.4 | 3.5 | |
| | 25 | 67 | 1.7 | 3.0 | + | + | - | 7.0 | - | 15 mo, once | 20.0 | 7.0 | |
| | 26 | 60 | 2.5 | 2.7 | + | + | - | 1.3 | - | 3.3 mo, 3 times | 29.6 | 29.6 | Relapse |
| Median | | 59.0 | 2.1 | 3.1 | N/A | N/A | N/A | 1.8 | N/A | N/A | 7.8 | 3.9 | - |
| Sum | | N/A | N/A | N/A | 22 | 11 | 7 | N/A | 9 | 8 | N/A | - | - |
*: Time from completion of radiotherapy to onset, †: Time from onset to disappearance of the first symptom, ‡: Times of relapses, ||: Time from onset to be free from symptoms, steroid and other medications, #: The practice of routine corticosteroid administration prior to 2001. Abbreviations: mo = months, N/A = not available
