# Supplementary material for: Steroid treatment increases the recurrence of radiation-induced organizing pneumonia after breast-conserving therapy
Source: Cancer Med. 2014 May 3;3(4):947–53. doi: 10.1002/cam4.255 (PMC4303162; doi:10.1002/cam4.255)
Supplement: Supplementary file 3 [file cam40003-0947-sd3.docx]

Table S2 Patient properties of Steroid and Nonsteroid groups

|  | | #Steroid (n=7) | #Nonsteroid (n=19) | P value |
| --- | --- | --- | --- | --- |
| Age | | 56 y.o.^§^ (SD: 12.0） | 60 y.o.^§^ (SD: 8.8） | 1.00* |
| Follow-up time | | 43.9 mo^§^ (SD: 57.8） | 41.2 mo^§^(SD: 31.7） | 0.86* |
| History of Endocrine therapy | | 3 (43%) | 15 (79%) | 0.15† |
| History of Chemotherapy | | 3 (43%) | 2 (11%) | 0.10† |
| cStage | I,  II-III | 6 (86%),  1 (14%) | 10 (53%)  9 (47%) | 0.19† |
| pStage | 0-I,  II-III | 2 (29%)  5 (71%) | 10 (53%)  9 (47%) | 0.39† |
| pT | pTis-1  pT2 | 4 (57%)  3 (43%) | 11 (58%)  8 (42%) | 1.00† |
| pN | pN0  pN1-2 | 4 (57%)  3 (43%) | 16 (84%)  3 (16%) | 0.29† |
| ER positive | | 3 (43%) | 15 (79%) | 0.15† |
| PR positive | | 2 (29%) | 13 (68%) | 0.095† |
| Her2 positive | | 2 (40%) | 2 (13%) | 0.23† |

Abbreviation: y.o., years old; SD, standard deviation; mo, months.

#: number of patients except for follow-up time and age, §: in median, *: Wilcoxon test, †: Fisher’s exact test.
