# Supplementary material for: Steroid treatment increases the recurrence of radiation-induced organizing pneumonia after breast-conserving therapy
Source: Cancer Med. 2014 May 3;3(4):947–53. doi: 10.1002/cam4.255 (PMC4303162; doi:10.1002/cam4.255)
Supplement: Supplementary file 4 [file cam40003-0947-sd4.docx]

Table S3 Evaluation of parameters and RIOP relapse

|  | | #Relapse + (n = 8) | #Relapse - (n = 18) | P value |
| --- | --- | --- | --- | --- |
| Steroid Administration | | 5 (71%) | 3 (16%) | 0.0138† |
| Age | | 58 y.o.^§^ | 59 y.o.^§^ | 0.80* |
| History of Endocrine therapy | | 5 (63%) | 13 (72%) | 0.67† |
| History of Chemotherapy | | 2 (25%) | 3 (17%) | 0.63† |
| cStage | I  II-III | 7 (88%)  1 (12%) | 9 (50%)  9 (50%) | 0.099† |
| pStage | 0-I  II-III | 4 (50%)  4 (50%) | 8 (44%)  10 (56%) | 1.00† |
| pT | pTis-1  pT2 | 6 (75%)  2 (25%) | 9 (50%)  9 (50%) | 0.39† |
| pN | pN0  pN1-2 | 5 (63%)  3 (37%) | 15 (83%)  3 (17%) | 0.33† |
| ER positive | | 6 (75%) | 12 (67%) | 1.00† |
| PR positive | | 4 (50%) | 11 (61%) | 0.68† |
| Her2 positive | | 1 (25%) | 3 (18%) | 1.00† |

Abbreviation: y.o., years old.

#: number of patients except for age, §: in median, *: Wilcoxon test, †: Fisher’s exact test.
